# Full-Scale Application of One-Stage Simultaneous Nitrification and Denitrification Coupled with Anammox Process for Treating Collagen Casing Wastewater

**DOI:** 10.3390/ijerph19105787

**Published:** 2022-05-10

**Authors:** Dayan Yu, Wenjie Zhang, Dunqiu Wang, Yue Jin

**Affiliations:** 1 Guangxi Key Laboratory of Environmental Pollution Control Theory and Technology, College of Environmental Science and Engineering, Guilin University of Technology, Guilin 541004, China; yudayanleo@163.com (D.Y.); 2010053@glut.edu.cn (W.Z.); 2 Guangxi Collaborative Innovation Center for Water Pollution Control and Water Safety in Karst Area, Guilin University of Technology, Guilin 541004, China; 3 College of Civil Engineering and Architecture, Guilin University of Technology, Guilin 541004, China

**Keywords:** low C/N, collagen casing wastewater, anaerobic ammoxidation, simultaneous nitrification and denitrification

## Abstract

The ammonia nitrogen (NH_4_^+^-N) concentration in the effluent released from the secondary sedimentation tank of the original collagen enteric coating wastewater treatment process considerably exceeded the Chinese effluent discharge standard. Therefore, a one-stage simultaneous nitrification and denitrification coupled with the anaerobic ammonia oxidation (SNDA) process was designed to terminally treat collagen enteric coating wastewater containing low COD/NH_4_^+^-N (C/N). The entire process start-up and NH_4_^+^-N loading (NLR) domestication phase was completed within two months. During the NLR domestication, the NH_4_^+^-N removal rate was more than 90% and its effluent concentration was less than 15 mg/L, guaranteeing that the NH_4_^+^-N in the subsequent effluent was within the standard value. The results of microbial diversity show that *Acinetobacter*, *Bacillus*, and other heterotrophic nitrification–aerobic denitrification bacteria, and anammox ammonia oxidation bacteria were the main functional bacteria at the genus level, exhibiting high denitrification performance. The one-stage SNDA process effectively and stably removed nitrogen; the treated sewage satisfied the national comprehensive wastewater discharge standard (GB8978-1996), effectively saving 30–40% of the floor area and reducing 67.6% of the additionally added alkali, wherein the system’s denitrifying bacteria compensated for some alkali consumed during the nitrification reaction.

## 1. Introduction

Guangxi Wuzhou Shenguan protein-casing factory specializes in casing production using cow leather as a raw material, and the acid hydrolysis method to prepare and extract collagen. The wastewater produced during casing processing contains copious amounts of animal protein, oil, and suspended solids. The ammonia nitrogen (NH_4_^+^-N) concentration in the effluent, after the original activated sludge treatment process, exceeded the Chinese effluent discharge standard. Its direct entry into the water body reduces the dissolved oxygen (DO) concentration, which directly affects the survival of aquatic organisms and causes water eutrophication [1]. Increased ammonia pollution from the food processing industry has heightened the need for nitrogen abatement strategies. In the traditional biological denitrification process, oxygen and carbon source requirements differ between aerobic nitrifying and anaerobic denitrifying bacteria, requiring the establishment and operation of separate aerobic and anaerobic reactors, resulting in complex processes and higher costs [2,3]. Heterotrophic nitrification–aerobic denitrification (HN-AD) bacteria have the advantages of both traditional nitrifying and denitrifying bacteria, and can perform simultaneous nitrification and denitrification (SND) reactions in the same reactor [2,4]. Under aerobic conditions, HN-AD bacteria reduce NO_3_^−^-N to nitrogen (N_2_), and the OH^−^ released during denitrification compensates for the alkali consumed during the nitrification reaction, which reduces the operation cost, shortens the denitrification cycle, and further achieves efficient sewage denitrification [5,6].

Most wastewaters contain insufficient organic carbon, resulting in incomplete denitrification and necessitating an additional carbon source, which increases the operation cost of sewage treatment. Anaerobic ammonia oxidation (ANAMMOX) is a new denitrification process, wherein anammox ammonia-oxidizing bacteria (AnAOB) can remove NH_4_^+^-N by oxidizing it to nitrogen with nitrite nitrogen (NO_2_^−^-N) as an electron acceptor [7,8,9]. Furthermore, AnAOB is a chemical autotrophic microorganism with CO_2_ as the main carbon source, and does not need the addition of external carbon sources to compensate for that consumed during denitrification. Moreover, the anammox process exhibits high nitrogen removal efficiency, which reduces the system’s aeration volume and operation cost, generates less excess sludge, and releases less greenhouse gas such as nitrous oxide (N_2_O) [10,11,12,13,14]. Therefore, the ANAMMOX process is considered to be a more efficient and energy-saving wastewater treatment method, and can treat wastewater with low C/N. During the practical application and development of the ANAMMOX process, a problem exists in that the raw wastewater lacks sufficient NO_2_^−^-N. Since the ratio of NO_2_^−^-N/NH_4_^+^-N during the ANAMMOX process is 1.32, NO_2_^−^-N must be sufficiently present during the treatment process to maintain stable reactor operation and to achieve the autotrophic denitrification of sewage [15,16]. 

Therefore, the simultaneous nitrification and denitrification process coupled with ANAMMOX can be used to treat wastewater with low C/N and insufficient organic carbon sources. The coupling of denitrification and ANAMMOX can effectively manage NO_2_^−^-N/NH_4_^+^-N fluctuation [17]. The synergy of nitrification, denitrification, and ANAMMOX in a single reactor has the advantages of low construction cost, small floor area, large volume load, and simple operation and better control of the unit [18,19].

Thus, by coupling simultaneous nitrification and denitrification with the ANAMMOX process (SNDA), we have successfully established a one-stage process to retreat the effluent discharged from the secondary sedimentation tank of the original sewage treatment unit of the protein casing plant. Furthermore, we have combined it with online monitoring and intelligent control to measure and regulate the contents of pH, DO, and NH_4_^+^-N in a timely manner, and to ensure stable operation of the one-stage SNDA process for its practical application, which further simplifies the wastewater treatment operation and management.

## 2. Materials and Methods

### 2.1. Project Introduction

**Figure 1:**
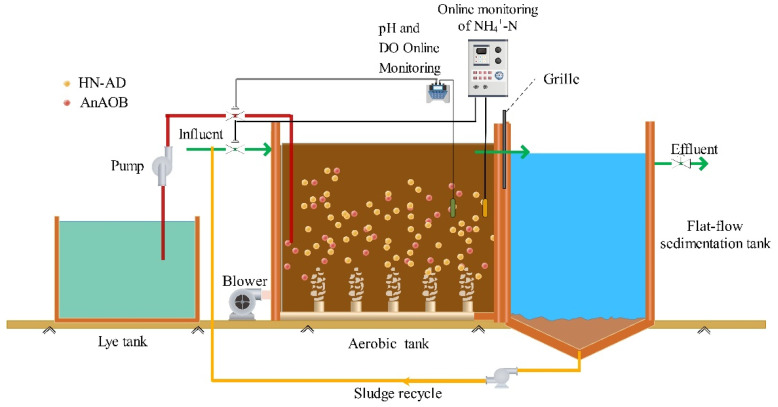
One-stage SNDA process flow diagram.

The one-stage SNDA process flow chart is shown in Figure 1, and contains an inlet pump, sludge circulating pump, aerobic tank, advection sedimentation tank, NH_4_^+^-N online monitoring, pH and DO online monitoring, lye tank and pump. The aerobic tank size is 40 m × 2.7 m × 2.45 m, with an effective volume of 216 m^3^. The sedimentation tank size is 40 m × 1.68 m × 2.45 m, with an effective volume of 131 m^3^. After being treated in the aerobic tank, the effluent from the secondary sedimentation tank flowed through the porous water distribution plate and baffle plate, and entered the advection sedimentation tank. After separating the mud water, the supernatant was discharged over the triangular weir. The sludge collected into the sludge bucket by the mud scraper was returned to the inlet of the aerobic tank via a circulating pump. An aeration device was placed at the bottom of the aerobic tank, and an NH_4_^+^-N online monitor, and pH and DO online monitor were installed at the end of the aerobic tank. The NH_4_^+^-N online monitor was used for operating and stopping the inlet pump, and regulating the NH_4_^+^-N concentration in the outlet water. When the NH_4_^+^-N value in the mixed liquid was lower than the lower limit of the NH_4_^+^-N online setting value, the inlet pump was run; when the NH_4_^+^-N value in the mixed liquid was higher than the upper limit of the NH_4_^+^-N online setting value, the inlet pump stopped. The alkali liquor pipe was evenly arranged along the body of the aerobic tank with 12 alkali liquor adding valves, and each alkali liquor valve was adjusted to ensure that the pH of the entire aerobic tank was maintained in the appropriate range. The pH online analyzer was used for operating and stopping the alkali pump. When the pH value in the mixed solution of the aerobic tank was lower than the lower limit of the pH online setting value, the alkali pump was run; when the pH value in the mixed solution of the aerobic tank was higher than the upper limit of the pH online setting value, the alkali pump stopped. One-stage SNDA records the changes in pH, DO, and NH_4_^+^-N content in real-time and regulates them according to the actual situation. During the process, the changes in pH, DO, and NH_4_^+^-N content were recorded in real-time and adjusted according to the actual situation.

### 2.2. Inoculated Sludge and Wastewater

The secondary sedimentation tank effluent of a sausage processing plant was the wastewater used in this study. Influenced by the original wastewater treatment process, the NH_4_^+^-N concentration in the secondary sedimentation tank effluent considerably exceeded the standard, necessitating its retreatment. The wastewater quality and effluent treatment design is presented in Table 1. The process start-up and operation were mainly divided into two stages; the specific operating conditions are shown in Table 2. In the initial stage of the start-up, the lower and upper limits of pH value online were set to 7.3 and 7.6, respectively; the lower and upper limits of NH_4_^+^-N online were set to 6 and 9 mg/L, respectively; and the flow of the inlet pump was set at 50 m^3^/h. During NLR adjustment, the lower and upper limits of pH value online were set to 7.2 and 7.4, respectively, by adding alkali (NaOH), while the lower and upper limits of NH_4_^+^-N online were increased accordingly. The DO concentration was controlled to be below 1.5 mg/L, and the inlet pump was adjusted to 25 m^3^/h to prolong the inlet time. The treatment mode varied from intermittent water inflow to continuous water inflow.

**Figure 2:**
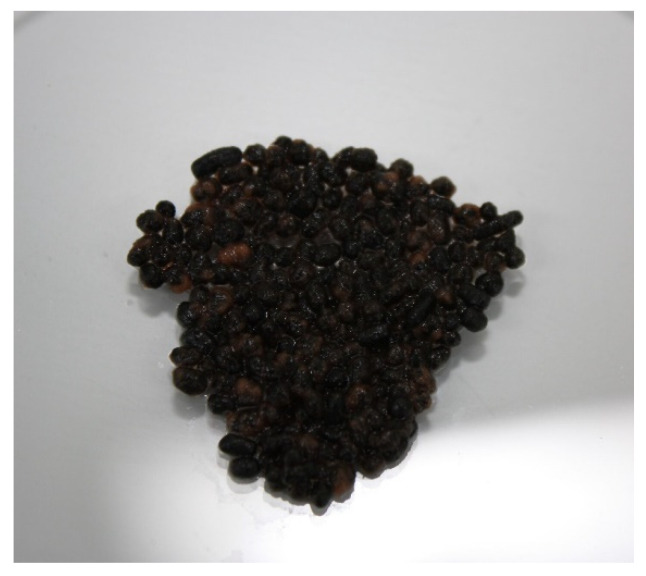
An AOB-loaded activated carbon packing.

The sludge to be inoculated was procured from the CASS tank of a sewage treatment plant. Activated sludge was added during the early stages of aerobic tank start-up, with MLSS of 2000~3000 mg/L. After one week of start-up and operation, 1% laboratory cultured AnAOB-activated carbon filler was added, as shown in Figure 2.

### 2.3. Analytical Method

The amount of nitrous nitrogen was determined using N-(1-naphthyl)-ethylenediamine spectrophotometry [20], and COD was determined using the rapid digestion method. The pH and DO were determined by the online monitoring system, and NH_4_^+^-N was determined online using the German WTW ammolyt plus 700 iq. During the entire experiment, the NH_4_^+^-N content, pH, and DO concentration in the effluent were monitored in real-time to monitor the reactor performance. For the convenience of calculation, all units of nitrogen and COD concentrations are in mg/L. The removal efficiency (%) of NH_4_^+^-N and COD is calculated using Formula (1) [21]:

(1) $$\frac{C\left(inf\right)-C\left(eff\right)}{C\left(inf\right)}\times 100\%,$$

where *C*(*inf*) and *C*(*eff*) are the concentration of NH_4_^+^-N and COD in the influent and effluent, respectively. OriginPro 2022 software (origin lab, Northampton, MA, USA) and SPSS 23.0 software were used for data analysis. The Pearson correlation test was performed to determine the correlation between the values of certain parameters. The results were considered to significantly differ at *p* < 0.05.

### 2.4. Microbial Diversity Analysis

To more intuitively understand the changes in the microbial community structure and diversity, high-throughput sequencing technology, which is a molecular biology technology, was used to determine the microbial community structure in the sludge samples before and after the reactor start-up. The sludge was sampled thrice after the sludge domestication stage (T1, T2, and T3), and was transported to sangon Bioengineering (Shanghai) Co., Ltd., (Shanghai, China) for performing high-throughput sequencing analysis to analyze the changes in microbial diversity and identify the dominant strains. The analysis method performed was according to that described by Liu et al. [7].

## 3. Results and Discussion

### 3.1. Start-Up Period

**Figure 3:**
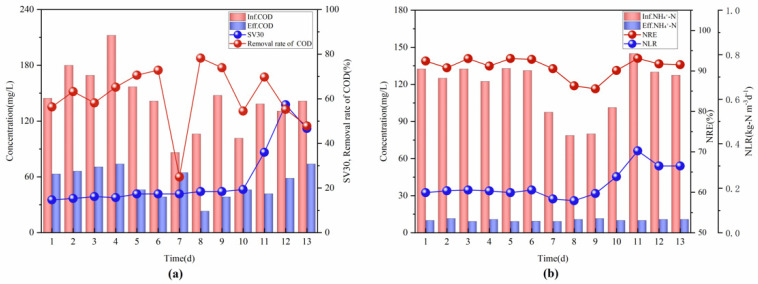
Variation in the indicators during the start-up period: (**a**) concentration of COD, COD removal rate, and SV30 in the inlet and outlet water; (**b**) concentration of NH_4_^+^-N, NH_4_^+^-N removal rate (NRE), and NLR in the inlet and outlet water.

It can be seen from Figure 3a that only nitrification and denitrification sludge was added to the aerobic tank during the first 7 days. This initial stage of the start-up contained less biomass, and SV30 was maintained at approximately 16.3%, with low nitrification and denitrification rates. Deng and Yang et al. found that acidic (pH < 5) or alkaline (pH > 10) conditions inhibit the growth and reproduction of HN-AD bacteria [22,23], and weak alkaline conditions are more conducive to their metabolism [24]. Therefore, the lower and upper limits of the pH online were set at 7.3 and 7.6, respectively, and the flow of the inlet pump was 50 m^3^/h. The lower and upper limits of NH_4_^+^-N online were set at 6 and 9 mg/L, respectively, by shortening the inlet time and prolonging the treatment time, and a low-NH_4_^+^-N load (NLR) was maintained in the aerobic tank for the growth of HN-AD bacteria. It can be seen from Figure 3b that at the initial stage of the start-up, the influent of NH_4_^+^-N was approximately 130 mg/L, the NLR was stable at approximately 0.18 kg-N m^−3^d^−1^, the effluent NH_4_^+^-N concentration was less than 10 mg/L, and the NRE was maintained at more than 90%. During this phase, a stable denitrification performance was observed, indicating that the HN-AD bacteria in the aerobic tank rapidly adapted to the surrounding environment and assisted in NH_4_^+^-N removal. Therefore, the activated carbon filler attached to AnAOB was added on the eighth day, and the NLR gradually increased to 0.366 kg-N m^−3^d^−1^. The added AnAOB competed with HN-AD for NH_4_^+^-N substrate. After several days of acclimation, NRE gradually increased and stabilized at more than 90%, indicating the successful establishment and start of the denitrification process. The DO concentration in the system was maintained below 1.5 mg/L; a lower DO concentration is conducive to the formation of an anoxic microenvironment in the activated sludge floc, which helps coordinate the anaerobic ammonia oxidation and aerobic denitrification processes, further strengthening the denitrification effect.

### 3.2. NLR Domestication Stage

**Figure 4:**
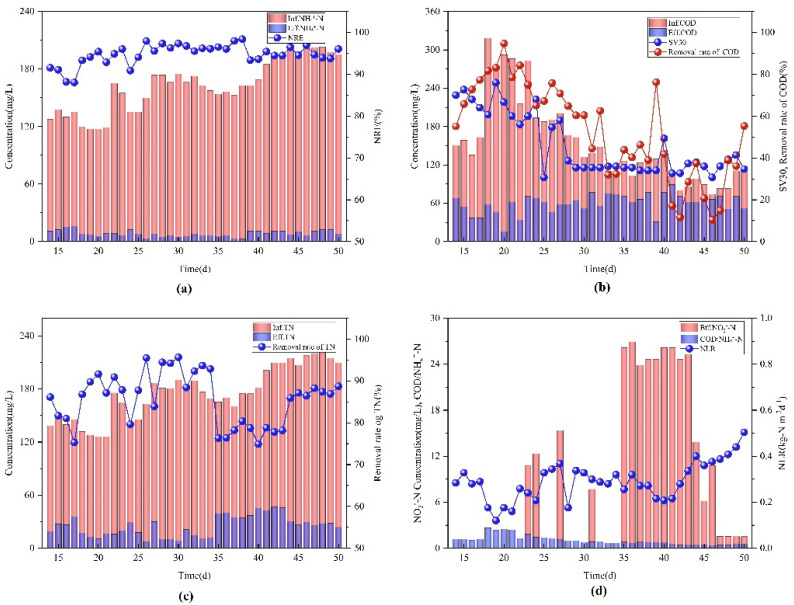
Changes in indexes during NLR domestication stage; (**a**) NH_4_^+^-N concentration and NRE in inlet and outlet water; (**b**) COD concentration in inlet and outlet water, COD removal rate and changes in SV30; (**c**) TN concentration and TN removal rate in inlet and outlet water; (**d**) effluent NO_2_^−^-N, NLR, and C/N.

After the start-up period, the one-stage SNDA system entered a stable operation period for a total of 37 days. During this period, the lower and upper limits of pH value online were set to 7.2 and 7.4, respectively; the lower and upper limits of NH_4_^+^-N online were set to 11 and 12 mg/L, respectively; and the inlet pump flow was adjusted to 25 m^3^/h. The inlet time was prolonged and the treatment time was shortened to improve the NLR in the aerobic tank. In Figure 4b, the influent COD changed in the range of 74~318.46 mg/L, with large fluctuations, which reflected the fluctuating characteristics of the effluent from the original secondary sedimentation tank; however, the effluent COD was maintained at approximately 60 mg/L, meeting China’s sewage discharge standards, which also demonstrates the ability of the fully mixed aeration system in resisting the impact load. Wang et al. also suggested that a complete mixed aeration system can provide a suitable growth environment for heterotrophic microorganisms including aerobic denitrifying bacteria, and most aerobic denitrifying bacteria have the capacity for both heteroxic nitrification and the metabolic degradation of organic matter [24]. During reactor operation, controlling the growth environment and operating conditions specific to the nitrifying bacteria, HN-AD bacteria, and AnAOB continuously eliminated several miscellaneous bacteria, which considerably reduced the number of microbial communities and the biomass, and reduced the SV30 from 70% to approximately 36.20%. Figure 4b shows that at higher influent COD concentration, the COD removal rate is relatively large, and that this decreases when the influent COD concentration is low. Carbon is the structural unit and a source of energy for microorganisms, whereas nitrogen can promote the synthesis of amino acids, proteins, and nucleic acids. Therefore, carbon and nitrogen are crucial for the growth and function of microbial cells [25]. Due to the low C/N in wastewater, aerobic heterotrophic bacteria that use organic matter as a nutrient substrate have insufficient nutrients available when the organic matter concentration is low, which renders the removal effect of COD unstable.

As observed in Figure 4d, NLR gradually decreased from day 14 to day 20 and reached the lowest value of 0.127 kg-N m^−3^d^−1^ on day 20. The influent during this period contained more white sediment and a higher COD concentration. After the influent returned to normal, NLR gradually increased. On the 26th day, the NLR increased and was maintained at 0.32 kg-N m^−3^d^−1^. HN-AD bacteria adapted to the surrounding environment, exhibiting increased activity, which gradually stabilized the denitrification rate. With the increase in influent concentration, the lower and upper limits of NH_4_^+^-N online were adjusted to 7 and 12 mg/L, respectively, on the 42nd day. The NLR gradually improved and accelerated the nitrogen removal efficiency in the aerobic tank. Furthermore, the upper limit of NH_4_^+^-N online was adjusted to 7~15 mg/L on the 48th day, which increased the NLR to ensure continuous influent supply on the 50th day. NLR is strongly positively correlated with NRE (*p* = 0.006 < 0.05); hence, improving the NLR is conducive to efficient and stable denitrification.

It can be seen from Figure 4a,d that HN-AD bacteria and AnAOB continued to grow and reproduce with NH_4_^+^-N as the substrate, and the NLR continued to increase from 0.127 kg-N m^−3^d^−1^ to 0.501 kg-N m^−3^d^−1^. The NRE was more than 94%, and the NH_4_^+^-N concentration in the effluent was less than 15 mg/L, indicating that the subsequent effluent did not exceed the standard limit for ammonia and nitrogen. Heterotrophic nitrifying and aerobic denitrifying bacteria can oxidize NH_4_^+^-N to NO_2_^−^-N or NO_3_^−^-N, and denitrify these products to N_2_O or N_2_ [26]. NO_2_^−^-N accumulation is often observed during the aerobic denitrification process [26]. Li et al. found that although HN-AD can achieve NO_3_^−^-N and NO_2_^−^-N denitrification, it cannot eliminate NO_2_^−^-N accumulation, which is consistent with the application results of this study [27]. However, the continuously high NH_4_^+^-N content in the influent inhibited NOB, and did not significantly change the effluent NO_3_^−^-N and NO_2_^−^-N contents, indicating that the increase in the NLR did not significantly affect the HN-AD bacteria and AnAOB in the system, and that the system was relatively stable. The uneven distribution of aeration devices easily produces a large proportion of localized anoxic microenvironment. Therefore, in this system, simultaneous nitrification and denitrification coupled with anaerobic ammonia oxidation can be effectively achieved by adjusting the NLR, DO, and pH. Compared with the traditional denitrification method, the one-stage SNDA process has the advantages of achieving nitrification, denitrification, and anaerobic ammonia oxidation simultaneously in one system, avoiding acidification of the nitrification system and balancing multifunctional microbial mixing culture, etc. It not only solves the problem of insufficient carbon source in the wastewater to be treated, but also effectively saves floor space and reduces the amount of alkali dosing in the system.

Synchronous nitrification–denitrification not only shortens the reaction time, but also reduces the consumption of organic carbon sources and simplifies the process, and HN-AD bacteria have higher growth rate, resistance to environmental stresses such as salinity, heavy metal ions and antibiotics than traditional autotrophic nitrifying bacteria, and are suitable for a wide range of wastewater treatments [25]. The anaerobic ammonia oxidation process does not require an additional carbon source and saves 60% in terms of energy consumption, but the process start-up time is longer and the enrichment of anaerobic ammonia-oxidizing bacteria is difficult [28]. Table 3 lists some of the practical applications of simultaneous nitrification–denitrification and anaerobic ammonia oxidation processes, from which it can be seen that the anaerobic ammonia oxidation process and simultaneous nitrification–denitrification process are commonly used for the denitrification of wastewater with a low carbon to nitrogen ratio and a high concentration of ammonia nitrogen, such as piggery wastewater, waste leachate wastewater, and coking industrial wastewater. Therefore, the one-stage SNDA process couples the simultaneous nitrification–denitrification and anaerobic ammonia oxidation processes for denitrification, in which heterotrophic denitrifying bacteria can also lift the inhibition of the anaerobic ammonia oxidation process by organic matter and dissolved oxygen, so the one-stage SNDA process in this study has a good application prospect in treating high-ammonia-nitrogen wastewater.

### 3.3. Microbial Analysis

**Figure 5:**
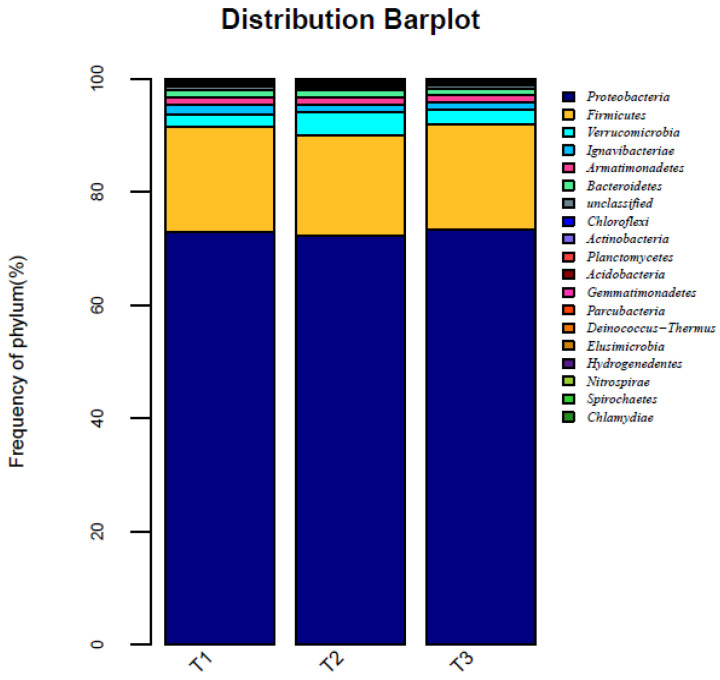
Changes in microbial community structure at the phylum level.

To determine the microbial community structure of one-stage SNDA process after startup and nitrogen load acclimation, the sludge samples (T1, T2, and T3) collected after the load test were analyzed. It can be seen from Figure 5 that the sludge samples T1, T2, and T3 mainly include five bacteria phyla: *Proteobacteria*, *Firmicutes*, *Verrucomicrobia*, *Ignavibacteriae,* and *Armatimonadetes*, among which *Proteobacteria* are the dominant phylum at the microbial level in the sludge [36]. In the activated sludge ecosystem, the stability of the system does not contribute to the change in microbial diversity; it mainly depends on the growth and change in functional bacteria [37]. Due to the changes in the inoculated sludge environment, some microorganisms die and collapse due to maladjustment. The COD breakdown in the cell provides a small amount of carbon source for denitrifying bacteria, and helps in the multiplication of HN-AD in the system. It can be seen from Table 4 that the rich microbial diversity in the sludge system after load acclimation establishes an efficient denitrification process. *Acinetobacter*, *Sporosarcina*, and *Planococcaceae* are the most abundant genera, of which HN-AD bacteria-related genera (such as *Acinetobacter* and *Bacillus*) are absolutely dominant, accounting for more than 70% of the total. Anaerobic ammonia oxidation and aerobic denitrification promote and compete with each other in the one-stage reaction system. Although both accept NH_4_^−^-N as the reaction substrate, AnAOB find it difficult to compete with HN-AD bacteria in terms of population number. Moreover, NO_2_^−^-N produced by aerobic denitrification can also provide electronic receptors for anaerobic ammonia oxidation. They are efficient and synergistic, thereby ensuring the efficient and stable denitrification performance of the system.

### 3.4. Engineering Benefit

**Figure 6:**
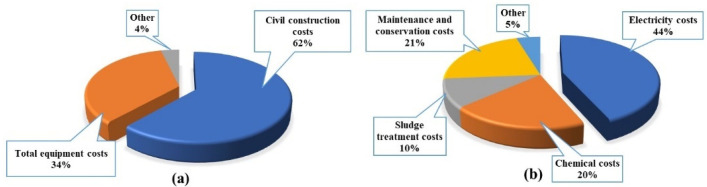
One-stage SNDA process cost analysis; (**a**) distribution of total project investment; (**b**) distribution of operating costs.

The one-stage SNDA process reduces the reactor volume by 30–40% and saves the capital construction cost. The total investment for the entire study mainly comprised civil engineering cost, total equipment cost, and others (Figure 6a), with a total cost of approximately 1.62 million yuan. Since the sewage treatment process adopts PLC automatic control operation, and the technicians of the original sewage treatment plant are responsible for the operation, the labor cost is ignored. Therefore, the operating cost mainly consisted of electricity, chemicals, repair and maintenance, sludge disposal, and other expenses (Figure 6b), totaling at RMB 0.449 per m^3^ (USD 0.07/m^3^, exchange rate USD 1 = RMB 6.36), based on 600 m^3^ of wastewater treated per day. Electricity was mainly generated by pumps and blowers, consuming approximately 20 kwh per day. Considering the price of RMB 0.7/kW·h (large industrial electricity), the electric charge was RMB 0.196 per m^3^ of wastewater treatment (USD 0.02/m^3^, exchange rate USD 1 = RMB 6.36), accounting for 44% of the total operating cost. The cost for chemical agents was largest for NaOH, with a daily requirement of 150 kg. Therefore, a chemical agent fee of RMB 0.089 is required for each m^3^ of wastewater treatment (USD 0.014/m^3^, exchange rate of USD 1 = RMB 6.36). The repairs and maintenance costs are calculated at 1.65% of the total construction cost, based on a conventional fixed asset formation rate of 80%, so the repair and maintenance cost is RMB 0.097 per m^3^ (USD 0.015/m^3^, exchange rate of USD 1 = RMB 6.36). Sludge disposal cost is RMB 0.046 per m^3^, accounting for 10% of the total operating cost. Other costs include sewage treatment for flushing various structures, pool surface, sludge treatment equipment water consumption, sewage, sludge test fees, greening fees, etc., totaling RMB 0.021 per m^3^ (USD 0.003/m^3^, exchange rate of USD 1 = RMB 6.36). Compared with the traditional nitrification and denitrification process, the electricity cost was reduced by 20%. The alkali produced during denitrification in the one-stage SNDA process can reduce the additional dosage of alkali for nitrification, which reduces the reagent cost by approximately 67.6%. Therefore, the one-stage SNDA process can replace the expensive nitrification–denitrification process.

## 4. Conclusions

The one-stage SNDA process effectively treated the effluent from the secondary sedimentation tank of the collagen casing plant containing low C/N. The COD removal rate varied with the change in influent COD concentration, but the effluent COD was maintained at approximately 60 mg/L, which satisfied China’s sewage discharge standard. The engineering application results indicate that by adjusting the NLR, continuous water inflow was achieved on the 50th day. The average TN removal rate was approximately 80%, and the NRE reached more than 94%, indicating that NH_4_^+^-N in the subsequent effluent did not exceed the standard. The stability of the system mainly depended on the growth and change in functional bacteria; the main functional bacteria in the nitrogen load acclimation stage were *Acinetobacter*, *Bacillus*, *Armatimonadetes*_gp5, and *Candidatus Kuenrnia*. The symbiosis of these dominant bacteria contributed to the simultaneous and efficient removal of NH_4_^+^-N and COD. In addition, combined with the online application of NH_4_^+^-N, pH, and DO monitoring, automatic control of the system was achieved, which simplified the operation and management of sewage treatment, thereby saving approximately 43% of the operation cost compared with that of the traditional nitrification and denitrification process. Moreover, the system reduced the volume of the traditional reactor by 30%, thereby decreasing the capital construction cost.

## Figures and Tables

**Table 1:** Wastewater quality and design effluent.

	Unit	Wastewater Quality	Post-Treatment Water Quality
NH_4_^+^-N	mg/L	79.08~208.3	15
COD	mg/L	76~368.5	100
NO_2_^−^-N	mg/L	<2	-
NO_3_^−^-N	mg/L	<2	-
pH	-	6.4~8.5	6~9

**Table 2:** Operating conditions at different stages.

Stage	Operation Days (d)	Inlet Pump (m^3^/h)	NLR (kg-N m^−3^d^−1^)	OLR (kg-COD m^−3^d^−1^)	pH Limits	NH_4_^+^-N Limits (mg/L)
Start-up Period	1–13	50	0.144~0.368	0.48~1.18	7.3~7.9	6~9
NLR Domestication Stage	14–51	25	0.127~0.501	0.21~0.88	7.2~7.4	11~12

**Table 3:** Selected practical applications of simultaneous nitrification–denitrification and anaerobic ammonia oxidation processes.

Seed Sludge	Wastewater	Reactor	Scale	Reference
*Acinetobacter* sp. T1	Real piggery wastewater	A2O	Full-scale	[29]
*A. faecalis* No.4 strains	Real piggery wastewater	Aerobic reactor	Lab-scale	[30]
*Acinetobacter junii* YB	High-strength ammonium wastewater (synthetic)	SBR	Lab-scale	[31]
*Kuenenia stuttgartiensis*	livestock manure digester liquor	-	-	[32]
Anaerobic ammonia oxidation seed sludge and activated sludge	Landfill leachate treatment	UFR	Lab-scale	[33]
Activated sludge	Synthetic coke-ovens wastewater	-	Lab-scale	[34]
Sludge from reactor treating a synthetic wastewater	High-strength optoelectronic industrial wastewater	SBR	Lab-scale	[35]

**Table 4:** Abundance of the main functional bacteria at the genus level.

Functional Bacteria	T1	T2	T3
*Acinetobacter*	68.32	67.20	69.01
*Sporosarcina*	5.23	5.22	5.03
*Planococcaceae*	4.36	3.91	4.41
*Bacillus*	2.92	2.63	2.81
*Armatimonadetes*_gp5	1.23	1.41	1.44
*Candidatus_Kuenrnia*	0.08	0.10	0.06
*Nitrosomonas*	0.05	0.05	0.04

## Data Availability

Not applicable.

## References

[1] Brase L., Sanders T., Dahnke K. (2018). Anthropogenic changes of nitrogen loads in a small river: External nutrient sources vs. internal turnover processes. Isot. Environ. Health Stud..

[2] Zhang Q., Liu Y., Ai G., Miao L., Zheng H., Liu Z. (2012). The characteristics of a novel heterotrophic nitrification-aerobic denitrification bacterium, *Bacillus methylotrophicus* strain L7. Bioresour. Technol..

[3] Zhao B., Tian M., An Q., Ye J., Guo J. (2017). Characteristics of a heterotrophic nitrogen removal bacterium and its potential application on treatment of ammonium-rich wastewater. Bioresour. Technol..

[4] Chen J., Gu S., Hao H., Chen J. (2016). Characteristics and metabolic pathway of *Alcaligenes* sp. TB for simultaneous heterotrophic nitrification-aerobic denitrification. Appl. Microbiol. Biotechnol..

[5] Lei X., Jia Y., Chen Y., Hu Y. (2019). Simultaneous nitrification and denitrification without nitrite accumulation by a novel isolated *Ochrobactrum anthropic* LJ81. Bioresour. Technol..

[6] Liu T., He X., Jia G., Xu J., Quan X., You S. (2020). Simultaneous nitrification and denitrification process using novel surface-modified suspended carriers for the treatment of real domestic wastewater. Chemosphere.

[7] Liu X., Jin Y., Zhang W. (2020). Effect of nitrite concentration on the growth and microbial diversity of anaerobic ammonia oxidation (anammox) sludge. Desalin. Water Treat..

[8] Ma B., Wang S., Cao S., Miao Y., Jia F., Du R., Peng Y. (2016). Biological nitrogen removal from sewage via anammox: Recent advances. Bioresour. Technol..

[9] Ma X., Liu X., Xiang B., Zhang W. (2019). Effect of Hydraulic Retention Time on Carbon Sequestration during the Two-Stage Anammox Process. Processes.

[10] Wen R., Wei Y., Zhang W. (2021). Recovery of nitrogen removal by N_2_H_4_ after nitrite inhibited anammox reaction. Global NEST J..

[11] Liu X., Wang D., Zhang W. (2020). Rapid start-up of anammox reactor using granular sludge supported on activated carbon. Global NEST J..

[12] Kuenen J.G. (2008). Anammox bacteria: From discovery to application. Nat. Rev. Microbiol..

[13] Jin Y., Zhang W. (2021). Nitrous oxide emissions from an anammox reactor from the startup to stable-running period. Desalin. Water Treat..

[14] Liu X., Wang H., Li H., Jin Y., Zhang W. (2019). Carbon sequestration pathway of inorganic carbon in partial nitrification sludge. Bioresour. Technol..

[15] Wei Y., Jin Y., Zhang W. (2020). Domestic Sewage Treatment Using a One-Stage ANAMMOX Process. Int. J. Environ. Res. Public Health.

[16] Wang H., Han J., Zhang W. (2019). Effects of NH_4_^+^-N and NO_2_^−^-N on carbon fixation in an anaerobic tor ammonium oxidation reactor. J. Environ. Manag..

[17] Ding S., Bao P., Wang B., Zhang Q., Peng Y. (2018). Long-term stable simultaneous partial nitrification, anammox and denitrification (SNAD) process treating real domestic sewage using suspended activated sludge. Chem. Eng. J..

[18] He Q., Song Q., Zhang S., Zhang W., Wang H. (2018). Simultaneous nitrification, denitrification and phosphorus removal in an aerobic granular sequencing batch reactor with mixed carbon sources: Reactor performance, extracellular polymeric substances and microbial successions. Chem. Eng. J..

[19] Chen H., Liu S., Yang F., Xue Y., Wang T. (2009). The development of simultaneous partial nitrification, ANAMMOX and denitrification (SNAD) process in a single reactor for nitrogen removal. Bioresour. Technol..

[20] Eaton A.D., Clesceri L.S., Greenberg A.E., Franson M.A.H. (1966). Standard methods for the examination of water and wastewater. Am. J. Public Health Nations Health.

[21] Bi Z., Zhang W., Song G., Huang Y. (2019). Iron-dependent nitrate reduction by anammox consortia in continuous-flow reactors: A novel prospective scheme for autotrophic nitrogen removal. Sci. Total Environ..

[22] Yang J., Wang Y., Chen H., Lyu Y.K. (2019). Ammonium removal characteristics of an acid-resistant bacterium *Acinetobacter* sp. JR1 from pharmaceutical wastewater capable of heterotrophic nitrification-aerobic denitrification. Bioresour. Technol..

[23] Deng M., Zhao X., Senbati Y., Song K., He X. (2021). Nitrogen removal by heterotrophic nitrifying and aerobic denitrifying bacterium *Pseudomonas* sp. DM02: Removal performance, mechanism and immobilized application for real aquaculture wastewater treatment. Bioresour. Technol..

[24] Wang S., Xu Q., Zhang G., Li Q. (2017). Operational Performance and Microbial Community Structure in a Completely Mixed Aeration System. Environ. Sci..

[25] Xi H., Zhou X., Arslan M., Luo Z.J., Wei J., Wu Z., El-Din M.G. (2022). Heterotrophic nitrification and aerobic denitrification process: Promising but a long way to go in the wastewater treatment. Sci. Total Environ..

[26] Zhou M., Ye H., Zhao X. (2014). Isolation and characterization of a novel heterotrophic nitrifying and aerobic denitrifying bacterium *Pseudomonas stutzeri* KTB for bioremediation of wastewater. Biotechnol. Bioprocess Eng..

[27] Li C., Yang J., Wang X., Wang E., Li B., He R., Yuan H. (2015). Removal of nitrogen by heterotrophic nitrification-aerobic denitrification of a phosphate accumulating bacterium *Pseudomonas stutzeri* YG-24. Bioresour. Technol..

[28] Wen R., Jin Y., Zhang W. (2020). Application of the anammox in china—A review. Int. J. Environ. Res. Public Health.

[29] Chen S., He S., Wu C., Du D. (2019). Characteristics of heterotrophic nitrification and aerobic denitrification bacterium *Acinetobacter* sp. t1 and its application for pig farm wastewater treatment—Sciencedirect. J. Biosci. Bioeng..

[30] Joo H.S., Hirai M., Shoda M. (2006). Piggery wastewater treatment using alcaligenes faecalis strain no. 4 with heterotrophic nitrification and aerobic denitrification. Water Res..

[31] Yang L., Ren Y.X., Liang X., Zhao S.Q., Wang J.P., Xia Z.H. (2015). Nitrogen removal characteristics of a heterotrophic nitrifier acinetobacter junii yb and its potential application for the treatment of high-strength nitrogenous wastewater. Bioresour. Technol..

[32] Jin Y., Zhang W. (2016). NaH_2_PO_4_ as pH buffer in an anaerobic ammonium oxidation (anammox) reactor treating high-strength livestock manure digester liquor. Desalin. Water Treat..

[33] Liang Z., Liu J. (2008). Landfill leachate treatment with a novel process: Anaerobic ammonium oxidation (Anammox) combined with soil infiltration system. J. Hazard. Mater..

[34] Toh S., Ashbolt N. (2002). Adaptation of anaerobic ammonium-oxidising consortium to synthetic coke-ovens wastewater. Appl. Microbiol. Biotechnol..

[35] Daverey A., Su S.H., Huang Y.T., Chen S.S., Sung S., Lin J.G. (2013). Partial nitrification and anammox process: A method for high strength optoelectronic industrial wastewater treatment. Water Res..

[36] Zhang T., Shao M.F., Ye L. (2012). 454 Pyrosequencing reveals bacterial diversity of activated sludge from 14 sewage treatment plants. ISME J..

[37] Zi Z., Bing T. (2015). Research progress in the microbial community and its functional characteristics in activated sludge systems. Ind. Water Treat..

